# Clinical experience with transcutaneous supraorbital nerve stimulation in patients with refractory migraine or with migraine and intolerance to topiramate: a prospective exploratory clinical study

**DOI:** 10.1186/s12883-017-0869-3

**Published:** 2017-05-18

**Authors:** Michail Vikelis, Emmanouil V. Dermitzakis, Konstantinos C. Spingos, Georgios G. Vasiliadis, George S. Vlachos, Evaggelia Kararizou

**Affiliations:** 1 Mediterraneo Hospital, Headache Clinic, Glyfada, Greece; 2 Glyfada Headache Clinic, Glyfada, Greece; 3 Geniki Kliniki Euromedica, Department of Neurology, Thessaloniki, Greece; 4 Corfu Headache Clinic, 13, Mitropolitou Methodiou str, 49100 Corfu, Greece; 5 Headache Outpatient Clinic, 1st Department of Neurology, National and Kapodistrian University of Athens, Athens, Greece

## Abstract

**Background:**

Migraine is included in the top-ten disabling diseases and conditions among the Western populations. Non-invasive neurostimulation, including the Cefaly® device, for the treatment of various types of pain is a relatively new field of interest. The aim of the present study was to explore the clinical experience with Cefaly® in a cohort of migraine patients previously refractory or intolerant to topiramate prophylaxis.

**Methods:**

A prospective, multi-center clinical study was performed in patients diagnosed with episodic or chronic migraine with a previous failure to topiramate treatment requiring prevention with Cefaly® according to the treating physician’s suggestion. A 1-month period of baseline observation was followed by a 3-month period of observation during the use of transcutaneous supraorbital nerve stimulation (t-SNS) with Cefaly® as the only preventive treatment.

**Results:**

A small but statistically significant decline was shown over time in the number of days with headache (HA), the number of days with HA with intensity ≥5/10, and the number of days with use of acute medication after 3 months (*p* < 0.001 for all of the three changes). Twenty-three patients (65.7%) expressed their satisfaction and intent to continue treatment with Cefaly®. Compliance was higher among satisfied subjects compared to non-satisfied subjects. None of the explored factors were significantly associated with the reason for the failure of topiramate.

**Conclusion:**

Three-months of preventive treatment for episodic or chronic migraine with t-SNS proved to be an effective, safe and well tolerated option for the treatment of patients with migraine who were intolerant or did not respond to topiramate.

**Trial registration:**

ClinicalTrials NCT03125525. Registered 21 April 2017.

**Electronic supplementary material:**

The online version of this article (doi:10.1186/s12883-017-0869-3) contains supplementary material, which is available to authorized users.

## Background

Migraine is ranked as the sixth most disabling condition, worldwide [1]. Presently, medications are the mainstream of migraine management; however, preventive treatment is often far from optimal [2]. Preventive treatment for migraine is usually considered when migraine pain is present more frequently than twice a week [3, 4].

Topiramate is currently the most commonly used first-line approved preventive medication for migraine [5]. With this being said, not all patients respond to preventive medications, due to either lack of efficacy or to adverse events. As a matter of fact, adherence to migraine preventive medications, including topiramate, may be insufficient. In a health insurance database based review, 70.2% of patients who initiated migraine prophylaxis with antiepileptics were reported to be non-adherent after 6 monhts [6]. Among preventive medication choices, patients are reported to adhere best, but not optimally to topiramate, with adverse events being the most common reason for topiramate discontinuation [7].

On the other hand, non-invasive neurostimulation is a relatively new field of interest for the treatment of various types of pain [8]. Clinical research in this field is active, as the recent technological advances allow for safe, convenient and ease by which to self-administer treatment sessions. Cefaly® electrically the supraorbital nerve in the forehead. The supraorbital nerve is a branch of the first trigeminal division. The trigeminovascular system has a well-known involvement in headache pain [9, 10]. Transcutaneous supraorbital nerve stimulation (t-SNS) with the Cefaly® (Cefaly® Technology sprl, Herstal, Belgium) device has proved to be a safe and efficient method for convenient self-delivered treatment sessions [11]. It has received approval for the prevention of episodic migraine by the American Food and Drug Administration and by the EU, including Greece, since early 2015 [12].

Although t-SNS use is spreading in Greece, it is not reimbursed by the social security system and in many cases it may be postponed until either a first line preventive medication fails to provide substantial relief or tolerability/safety issues ensue.

The aim of the present study was to explore and share the clinical experience with Cefaly® in a cohort of migraine patients previously refractory or intolerant to preventive treatment with topiramate, as this is a common situation in clinical practice. Additionally, we specifically explored whether the reason for the discontinuation of topiramate is correlated with the outcome of Cefaly® treatment. To the best of the authors’ knowledge, no similar study has been published so far.

## Methods

This was an exploratory prospective multicenter clinical study conducted in accordance with the principles of the Helsinki Declaration and approved by the principal investigator’s (MV) Institutional Review Board. This study was done to explore the efficiency and safety of Cefaly® in migraine prevention in a population of patients previously refractory or intolerant to topiramate.

Participants to be treated with Cefaly® were enrolled from 2 private headache clinics, located in Athens (Glyfada area) and Thessaloniki, the first and second largest cities of Greece, respectively. Patients were diagnosed with episodic or chronic migraine, needed preventive treatment according to the treating physician’s opinion, and they had not responded to previous topiramate treatment, either due to inefficacy or due to intolerability or safety issues. In order to consider topiramate as failed due to inefficacy, a dose of 100 mg/day for at least 3 months was required to have been received. Topiramate was considered as failed due to intolerability in any case a patient had decided to stop use of topiramate due to an adverse event regardless of its nature or severity. Patients had to have stopped topiramate at least 3 months prior to starting treatment with Cefaly®.

Both episodic and chronic (≥15 days of headache per month) migraine patients, according to the International Classification of Headache Disorders 3rd edition-beta version (ICHD IIIβ), were included [13]. Upon enrolment and after giving consent to participate in the study, demographics and clinical data were captured, including the reason for topiramate discontinuation. Patients were then provided with a headache (HA) diary to be self-completed over the course of the study including questions about occurrence of HA, peak intensity level on a 0–10 numerical scale, number of acute medication doses and any adverse event. A 1-month baseline observation period was followed by a 3-month active treatment period with Cefaly® as the only preventive treatment. During the active treatment period, compliance (days the device was used as recommended, e.g. 1 full session each day) was also recorded.

The European version of Cefaly® includes three stimulation programs; one for acute migraine relief and two programs to be implemented in daily 20-min sessions, one for migraine prevention and one for relaxation. In our study, Cefaly® was to be used based upon the protocol of the approval study of the device, in which it is used once every day on the migraine prevention program [14].

At their last evaluation, patients answered two additional questions regarding their total subjective satisfaction from treatment with t-SNS. The first question (“Are you satisfied with Cefaly® and wish to continue the treatment?”) was aimed to access overall satisfaction from t-SNS treatment and the will to continue treatment, which was the primary evaluation of our study. The second question (“Did you encounter technical issues with the device?”) was aimed to access any problems or technical difficulties arising from the use of the device.

Changes in total headache days, number of HA days with intensity ≥5/10 and days with acute medication use were analysed from baseline to the last observation or Month-3 of active treatment.

Additionally, the Fisher’s Exact test and the Mann-Whitney test were performed to test the association between satisfaction from Cefaly® and all other factors, including the reason to discontinue topiramate. In order to evaluate longitudinal changes over time for the number of headache days, the number of medication doses or the number of adverse events (AEs), Linear Mixed Models were performed, with patients modelled as a random effect, time (study month), group (e.g. whether they were satisfied or not), and their interaction modelled as fixed effects.

Statistical significance was set to the observed level of 5%. All statistical analyses were performed using STATA v.13.

## Results

From a total of 37 patients, 35 (F:31; age 22-62 yr.; median 45) were our intention-to-treat (ITT) population (2 dropped out before using Cefaly®), 32 were present for their last scheduled evaluation at 3 months and 27 (81.8%) had completed the 3-months of treatment with Cefaly®.

We recorded significant changes in headache days, headache days with pain intensity ≥5/10 and use of analgesics in patients under treatment with Cefaly® from 4 weeks baseline to the 4 weeks prior to last observation or at Month-3 (by a median of 2 days, *p* < 0.001; 2 days, *p* < 0.001; 4 doses, *p* < 0.001, respectively).

Twenty three patients out of 35 (65.7%) expressed their satisfaction and intent to continue treatment with Cefaly®. Trial completion was significantly higher among the satisfied subpopulation (21/27 [77.8%] vs. 6/27 [22.2%] in the non-satisfied subpopulation; *p* = 0.001). Compliance, expressed as the median [min-max] of total days the device was used as recommended, was 86/90 days, higher among satisfied subjects compared to non-satisfied (87 [78–90] days vs. 72.5 [30–90] days, respectively) (Table 1).

**Table 1:** Satisfaction and compliance at the end of 3-months trial of Cefaly®

	*N* (%)
Satisfaction-intent to continuation	
Yes	23 (65,7%)
No	12 (34,3%)
Completed 3 months trial	
Yes	27 (81,8%)
No	6 (18,2%)

The number of participants with a 50% or greater reduction in frequency of headaches at the end of the study vs. the baseline (responder rate) was 1/35, 0/32 and 1/31, for the 1st, 2nd and 3rd month of active treatment, respectively.

**Fig. 1:**
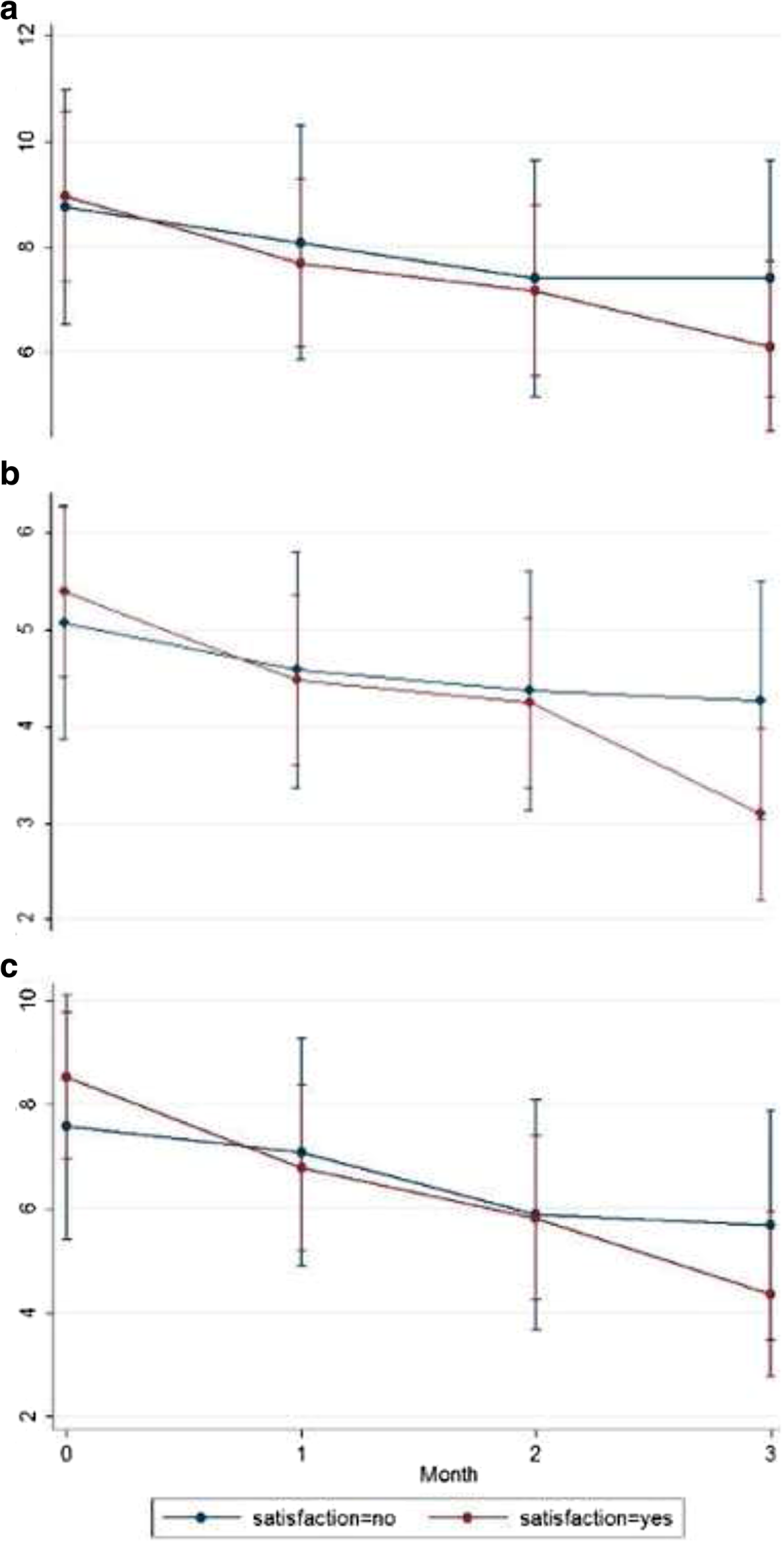
Number of headache days (**a**,) number of headache days with intensity ≥5/10 (**b**) and number of days with acute medication use (**c**) by patient satisfaction over the 3-months active treatment period with Cefaly®

**Fig. 2:**
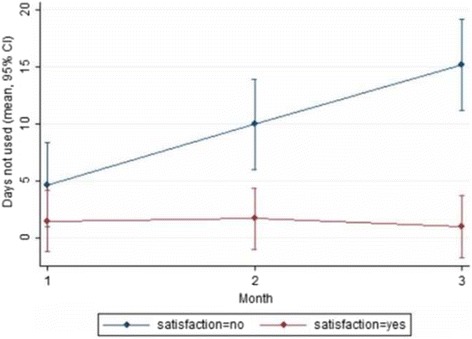
Number of days the device was not used by patient satisfaction over the 3-months active treatment period with Cefaly®

At baseline, mean (SD) number of days with HA was 8.9 (4.7) with a mean of 5.3 (2.4) showing peak intensity ≥5/10. Patients used acute medication for a mean of 8.2 (4.6) days (e.g. paracetamol, non-steroidal anti-inflammatories, triptanes etc.). On the last evaluation (the 3rd month of the trial), the respective means (SD) were 6.3 (3.5), 4.3 (1.8) and 4.4 (3.3). Results from longitudinal analysis with Linear Mixed Models showed significant decline over time in the number of days with HA and the number of days with HA with intensity ≥5/10 after 3 months (*p* = 0.007 and *p* < 0.001, respectively), the number of days with acute medication after 1 and 3 months (*p* = 0.022 and *p* < 0.001, respectively) and the days not used after 2 and 3 months (*p* = 0.043 and *p* < 0.001, respectively), for those who expressed satisfaction from Cefaly® in comparison to those who did not express satisfaction (Figs. 1 and 2).

The patients with chronic migraine (6 in our cohort) had similar percentages of satisfaction/intent to continue treatment compared to the patients with episodic migraine (66.7% vs. 65.5% respectively).

Four out of 35 patients (12.1%) experienced technical issues, which were easily resolved through on-call support. Twelve out of 35 (34.3%) patients reported an AE. All twelve reported AEs were unpleasant local paresthesias of mild intensity and they tended to decrease with time. No significant interaction of satisfaction with time was observed in regard to the number of AEs.

Among the intention-to-treat population, AEs were the primary reason of topiramate failure (57.1%). None of the explored factors was significantly associated with the reason for topiramate failure.

## Discussion

In the present study we explored the efficiency and safety of Cefaly® used in episodic and chronic migraine prevention, after a failed trial of topiramate. Significant changes in headache days, headache days with headaches ≥5/10, use of analgesics, a high percentage of satisfaction and intent to continue treatment with Cefaly®, a poor responder rate and a very low percentage of AEs and technical issues were found in patients under active treatment with Cefaly from the 4 weeks of baseline period (Month-1) to the 4 weeks prior to the last observation or the 3rd month of active treatment.

A trial dose of 200 mg topiramate, as suggested by some experts but not all, was not offered to our patients. This was due to a recent meta-analysis of three studies that had included more than one dose of topiramate and suggested that the 200 mg dose is no more effective than the 100 mg dose [15]. Up to the present (March 2017), Cefaly® is not reimburshed by insurance schedules as a migraine treatment in Greece though the recently approved (2015) device is frequently used after a failed trial of medical treatment - commonly topiramate. Therefore, this criterion for inclusion may represent not only the general clinical recommendations but also the actual clinical practice needs as well, in Greece and elsewhere.

An important limitation of this study was the lack of a control group and the small size of our sample. Future studies should randomise participants to a control group where stimulation current is thought to be too low to be efficacious vs. standard current. Or compare participants to 3 or 6 months receiving topiramate vs. stimulation use, in an open manner for those who have not tried topiramate. Headache location is not used as a criterion for management decisions in migraine and changes in location within the same attack or between attacks is common among migrainous patients. Because of this, data regarding location was not collected or analyzed in this small exploratory study [3]. However, future studies or meta-analyses might explore the response or the adverse events of Cefaly® in patients differentially grouped according to the usual location of their headache pain.

A comparable percentage (70.59%) of satisfied patients was found in the verum group of the prospective, multicentre, double-blinded, randomized and sham-controlled PREvention of MIgraine using CEfaly® (PREMICE) study, which enrolled migraine patients with at least 2 attacks per month [16]. In another recent survey, 46.6% of 2313 renters of Cefaly® chose to return the device, which implied that they were not satisfied [11]. That leaves a 53.4% of satisfied patients. The difference might be attributed, among other reasons, to the different selection criteria in the survey, where patients were included on the basis of their triptan use for their headaches [11].

At baseline, the mean (SD) number of days with HA, which was one of our measures of efficacy, was 8.9 (4.7) while a mean of 5.3 (2.4) of those HAs showed peak intensity ≥5/10, which was one of our measures of efficacy. Regarding our third measure of efficacy, patients used acute medication for a mean of 8.2 (4.6) days (e.g. paracetamol, NSAIDS, triptanes etc.). On the last evaluation (after the 3rd active treatment month of the trial), the means (SD) for those measures were 6.3 (3.5), 4.3 (1.8) and 4.4 (3.3), respectively. Regarding the days with HA, these findings represent a reduction of 29.2%. In PREMICE study, patients in the verum group showed a comparable reduction of 32.3% in the days with HA [16]. Regarding acute medication use, a reduction of 46.3% was noted in the present study, comparable to the respective 36.7% in the PREMICE study [16].

Results from longitudinal analysis with Linear Mixed Models showed statistically significant decline over time in the following: the number of days with HA and the number of days with HA with intensity ≥5/10 after 3 months (*p* = 0.007 and *p* < 0.001 respectively), the number of days with use of acute medication after 1 and 3 months (*p* = 0.022 and *p* < 0.001 respectively), the number of days the device was not used after 2 and 3 months (*p* = 0.043 and *p* < 0.001 respectively) and finally, for those who expressed satisfaction in comparison to those who did not express satisfaction (Figs. 1 and 2). This was an expected finding as it confirms that patients were satisfied when the device helped them. It also emphasizes the importance of a full 3-month trial of t-SNS before determining efficacy, as it has been suggested by the researchers of the PREMICE study, that the maximum reduction in migraine frequency occurred only after 3 months of treatment with Cefaly® [16].

The present study, due to the small size of our sample, does not qualify for any vigorous exploration of safety with Cefaly®. Nevertheless, around 1 in 3 patients mentioned an AE. This may seem contrasting to a much lower percentage of 4.3% for AEs in the aforementioned survey [11]. However, in our own definition for AEs we included any unpleasant local paresthesia of any intensity intending to explore the whole range of events during the Cefaly® trial.

Among our intention-to-treat population, AEs were the primary reason for topiramate failure (57.1%). In the present study, we specifically aimed to determine whether the primary reason for the failure of topiramate – lack of efficacy or AEs – might be related to the possible future response to Cefaly®. No factors of response to Cefaly® were significantly associated with the reason for topiramate failure. The absence of time correlation between satisfaction and AEs suggests that AEs of topiramate, as a reason for its discontinuation, could be a more organic and not individualised perceptional factor compared to the seemingly unaffected AEs and non-satisfaction from Cefaly®.

The subgroup of 6 patients with chronic migraine from our cohort showed a similar profile of satisfaction/intent to continue treatment compared to the patients with episodic migraine (66.7% vs. 65.5% respectively). In an addendum to the PREMICE study [16], an increased effect size in relation to the frequency of HAs is reported, suggesting a possibly more favorable response in this subgroup with debilitating migraine, which is something that we were unable to confirm.

## Conclusions

In conclusion, 3-months of preventive treatment for episodic and chronic migraine with t-SNS (Cefaly®) proved to be an effective, safe and well tolerated treatment option in patients having failed to respond to topiramate due to either lack of efficacy or tolerability/safety issues. Further studies are needed, leading eventually to a definitive phase-3 clinical trial, with a long-term follow-up of at least 1 year, as well as an economic evaluation, before treatment with Cefaly® is widely adopted.

## Additional file

Additional file 1:
**Suppl database.** Clinical experience with transcutaneous. (XLS 58 kb)

## Abbreviations

AE: Adverse events
EU: European Union
HA: Headache
